# Short‐Term Outcomes of Advanced Pneumatic Compression Device Versus Usual Care Therapy for Head and Neck Cancer‐Related Lymphedema: A Multi‐Site Randomized Clinical Trial

**DOI:** 10.1002/hed.70155

**Published:** 2026-01-23

**Authors:** Barbara A. Murphy, Derek K. Smith, Cristina M. Kline‐Quiroz, Katrina M. Jensen, Ammar W. Sukari, Mihir K. Bhayani, Vikas Mehta, Harry Quon, Jennifer L. Shah, Christopher D. Willey, Neal E. Dunlap, Hoon K. Lee, Joseph M. Aulino, Sheila H. Ridner

**Affiliations:** ^1^ Division of Hematology and Oncology Vanderbilt University Medical Center Nashville Tennessee USA; ^2^ Division of Biostatistics and Computational Biology University of Iowa College of Dentistry Iowa City Iowa USA; ^3^ Department of Physical Medicine and Rehabilitation Vanderbilt University Medical Center Nashville Tennessee USA; ^4^ Advanced Head & Neck Rehabilitation Center of Texas Fort Worth Texas USA; ^5^ Department of Otolaryngology‐Head and Neck Surgery Karmanos Cancer Institute Detroit Michigan USA; ^6^ Department of Otolaryngology‐Head and Neck Surgery Rush University Medical Center Chicago Illinois USA; ^7^ Department of Otorhinolaryngology‐Head and Neck Surgery Montefiore Medical Center Bronx New York USA; ^8^ Department of Radiation Oncology and Molecular Radiation Sciences Johns Hopkins Medical Center Baltimore Maryland USA; ^9^ Department of Radiation Oncology University of Michigan Ann Arbor Michigan USA; ^10^ Department of Radiation Oncology University of Alabama Birmingham Birmingham Alabama USA; ^11^ Department of Radiation Oncology, University of Louisville Research Foundation Inc. University of Louisville Louisville Kentucky USA; ^12^ Radiation Oncology Richmond University Medical Center Staten Island New York USA; ^13^ Department of Radiology Vanderbilt University Medical Center Nashville Tennessee USA; ^14^ School of Nursing Vanderbilt University Nashville Tennessee USA

**Keywords:** advanced pneumatic compression device, head and neck, lymphedema, short‐term outcomes, usual care lymphedema therapy

## Abstract

**Background:**

Two‐month outcomes of advanced pneumatic compression device (APCD) and usual care (UC) in Head and Neck Cancer survivors with previously untreated lymphedema were compared.

**Methods:**

Participants in this multisite, randomized clinical trial were randomized to APCD or UC. The primary endpoint was severity of lymphedema symptoms. Secondary endpoints were anatomical lymphedema changes, biopsychosocial outcomes, and barriers to care.

**Results:**

Two hundred thirty‐six participants were enrolled (119 APCD, 117 UC). Analysis was intention‐to‐treat. Lymphedema‐associated symptom burden measured using the VHNSS and LSIDS was improved to a similar degree in both groups. APCD demonstrated a statistically significant improvement in external soft tissue swelling assessed by digital photography. No difference in CT imaging measures of lymphedema was noted. UC participants experienced barriers to care.

**Conclusions:**

APCD is an effective treatment for lymphedema in HNCS. The APCD addresses clinically significant barriers to therapist guided treatment. A hybrid approach may be complementary and optimize patient outcomes.

**Trial Registration:** NCT04797390.

## 1 Introduction

Approximately 71 100 Americans were projected to develop head and neck cancer (HNC) in 2024 [1]. Survival rates are high and many are destined to live with the side effects from cancer and its therapy [2, 3, 4, 5]. A common, but often under recognized, effect of HNC and its treatment is secondary lymphedema [4, 5, 6, 7]. Lymphedema manifests as soft tissue swelling which may involve external structures (e.g., face and neck) and internal structures (e.g., larynx and pharynx) [6]. External lymphedema can lead to skin changes, pain, discomfort, stiffness, and decreased range of motion [2, 5, 7]. Involvement of internal structures frequently produces functional deficits such as dysphagia, altered speech, and shortness of breath [7, 8]. Without early identification and timely therapy, lymphedematous soft tissues can become fibrotic and contracted resulting in profound function loss and disability [8].

The cornerstone of Usual Care lymphedema management is conducted in two phases: Phase 1 centers on Therapist Guided Lymphedema Treatment (TGLT), and Phase 2 addresses long term lymphedema home self‐care [9]. During Phase 1, management strategies include manual lymph drainage (MLD), use of compression garments or bandages, exercises for stretching and strengthening, and skin care. Patients are also taught critical self‐care techniques that must be performed during life‐long Phase 2 self‐care. Unfortunately, patients often fail to receive standard lymphedema treatment due to system and patient related barriers [7, 10, 11, 12, 13]. Effective methods for addressing barriers to lymphedema care are therefore needed.

Published studies have demonstrated that the use of an advanced pneumatic compression device (APCD) for management of lymphedema in HNC survivors (HNCS) is feasible and effective [14, 15]. To confirm these results, earlier we conducted a randomized wait‐list controlled pilot study comparing an APCD to control in HNCS (*N* = 49) with previously treated lymphedema. Statically significant and clinically meaningful outcomes were: [16] improvement in perceived ability to control lymphedema (*p* = 0.003), decreased visible external swelling (front view *p* < 0.001, right view *p* = 0.004, left view *p* = 0.005), reduced soft tissue symptoms (e.g., heaviness, tightness, *p* = 0.0008), and reduced neurological symptoms (tingling, pins, and needles, *p* = 0.047). Thus, the APCD demonstrated efficacy as second‐line therapy for HNCS with residual or recurrent lymphedema. Therefore, we chose to further study the ACPD in HNCS with treatment naïve lymphedema.

We conducted a Randomized Clinical Trial in HNCS with symptomatic, treatment naïve lymphedema assessing efficacy of two interventions: Usual Care and an APCD. Outcomes were evaluated using patient reported, clinician reported, and imaging measures. Herein, we report the findings after 2 months (short term outcomes).

## 2 Materials and Methods

### 2.1 Study Design and Setting

This open label, multi‐site, stratified, randomized, effectiveness trial included a combination of 10 academic and community sites. Institutional Review Board and Scientific Review Board approval were obtained per institutional protocol prior to recruitment. Clinicaltrials.gov NC‐T#04797390.

### 2.2 Procedures

**FIGURE 1:**
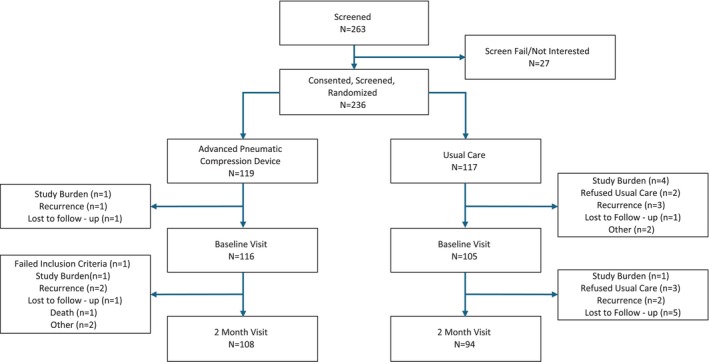
Consort diagram. [Color figure can be viewed at wileyonlinelibrary.com]

Site investigators and staff were trained by the study PI's on recruitment and data collection methods. Potentially eligible participants were identified based on medical record review (Figure 1). Staff obtained informed consent prior to any study specific screening assessments. Eligible individuals were stratified by site and randomized (1:1) to either an APCD or Usual Care.

### 2.3 Eligibility Criteria

Eligibility criteria included: age ≥ 18 years, pathologically confirmed cancer of the head and neck (larynx, pharynx, oral cavity, paranasal sinuses, major salivary glands, or unknown primary), completed curative intent therapy, no evidence of cancer at the time of study screening, and English speaking. In addition, eligible participants were diagnosed with either internal or external HNC associated lymphedema and had at least one core lymphedema associated symptom with a severity of ≥ 4 out of 10 [8, 17, 18]. Exclusion criteria included: previously treated head and neck lymphedema, facial infections, known carotid sinus hypersensitivity syndrome, symptomatic carotid artery disease, and internal jugular thrombosis within 3 months prior to consent.

### 2.4 Study Groups

ACPD Group: The APCD (Flexitouch Plus, Tactile Medical, Minneapolis, MN) was physician ordered as a 32‐min normal pressure treatment to be utilized daily for the duration of the study. A company‐trained representative conducted a therapy initiation visit during which participants were fitted for the pneumatic garments and taught donning and doffing techniques and instructed in controller operation. Participants were required to demonstrate fidelity to operational procedures of the APCD. Standardized educational materials and access to a digital application were provided [19]. The app includes educational material and a tool for tracking lymphedema symptoms and treatment sessions. No adjunctive therapies or routine follow‐up was included.

Usual Care Group: Participants were referred for TGLT per institutional procedures. We anticipated variability in Usual Care with regards to accessibility, assessment tools and techniques, treatment recommendations, implementation, patient adherence, enactment, and follow‐up [20]. No attempt was made to influence or modify Usual Care. Therapy notes were collected at the end of treatment for review and analysis (to be reported separately).

### 2.5 Outcome Measures

All PRO and CRO measures have been used in this population and are valid and reliable [4, 16, 17, 18, 19, 20, 21, 22, 23, 24, 25]. Patient reported outcome (PRO) measures were collected from participants either online via IMed net [21] or using paper forms. Clinician reported outcome (CRO) measures were captured in‐person by unblinded site investigators or trained study staff. Digital photographs and radiographic images were assessed by blinded central reviewers.

Except for imaging, outcome measures were completed at baseline, 2, 4, and 6 months. Imaging was captured at baseline, 2, and 6 months. The initial protocol required collection of baseline data within 7 days prior to the initial APCD or TGLT consultation. A protocol amendment was required due to the observed protracted interval between the referral and initiation of TGLT for some participants. The amendment stipulated that baseline measures would be collected 6 weeks after screening if the TGLT consultation was not completed by that time.

#### 2.5.1 PRO Measures

Participants completed six PROs; herein we report on two self‐report surveys that have been used extensively to assess symptom burden in this population. The Vanderbilt Head and Neck Symptom Survey plus General Symptom Survey (VHNSS plus GSS) measured head and neck cancer treatment related symptoms and functional impairment (61 items) [17]. The Lymphedema Symptom Intensity and Distress Survey—Head and Neck (LSIDS‐H&N v2.0) captured lymphedema specific symptoms (31‐items, seven subscales) [18]. The results of PROs measuring quality of life, body image, activity level, dietary intake, and lymphedema self‐management will be reported separately.

#### 2.5.2 CRO Measures

Internal lymphedema was assessed endoscopically with direct or indirect visualization of the upper aerodigestive tract and documented using the Modified Patterson Scale [22, 23].

External lymphedema was assessed by: (1) physical examination of the head and neck and documented using the Head and Neck Cancer Related Lymphedema and Fibrosis Grading (HN‐LEFG) criteria [25]; and (2) digital photographs (frontal, and left and right profiles) with a grid overlay [16]. The outcome measures for the HN‐LEFG included the number of sites involved with lymphedema (0 to a maximum of nine sites) and the total severity score, which is the summation of the severity score (one‐ mild, two‐moderate, three‐ severe) at each site (0 to maximum of 27).

#### 2.5.3 Imaging

CT images were scored using the CT Lymphedema and Fibrosis Assessment Tool (CT‐LEFAT) [24].

#### 2.5.4 Adherence and Adverse Events

Study staff interviewed participants every 2 weeks either in person or via telephone to assess for adherence and adverse events using a standardized questionnaire.

### 2.6 Statistical Analysis

All analyses reported in this manuscript were conducted using the Intention to Treat Principle with the caveat that missing outcome data was not imputed, so those missing 2 month data were excluded from the analysis. Statistical power for this study was based on a previous trial in participants with recurrent/residual lymphedema which had positive results for the APCD across six PRO subscales measuring lymphedema associated symptoms and functional impairment [16]. For these six subscales, observed differences ranged from 0.57 to 2.2 units with standard deviations ranging from 0.83 to 3.53 in individual groups. Based on these preliminary findings, it was determined that the planned study size of 250 was adequate to achieve power > 80% for each of the six PRO subscales and far higher for most. Accrual to the study was stopped at a prespecified time limit.

Continuous study variables were summarized as a mean (standard deviation) and categorical variables reported as number (%). All PRO measures were analyzed using a mixed‐effects regression with model covariates including the baseline value and treatment assignment with a random effect for study site. All comparisons were conducted at the alpha = 0.05 level.

## 3 Results

### 3.1 Sample Characteristics

Two hundred thirty‐six participants were randomized via a permuted block design to either Usual Care (*n* = 117) or APCD (*n* = 119)(Figure 1). The study groups were well balanced according to age, race, ethnicity, sex, tobacco usage, alcohol consumption, BMI, and rural versus urban locale (Table 1). In addition, no statistically significant differences were noted for cancer or treatment‐related factors. The extent of baseline lymphedema was similar between study groups as confirmed by the lack of significant differences on the Modified Patterson Scale, HN‐LEFG, blinded rating of digital photos, and blinded measurements of CT data.

**TABLE 1:** Baseline sociodemographic and clinical characteristics.

	Usual care (*N* = 117)	APCD (*N* = 119)	Overall (*N* = 236)	*p*
Age				
Mean (SD)	60.0 (11.0)	62.7 (9.78)	61.8 (10.9)	0.207
Median (min, max)	60.0 (20.0–94.0)	63.0 (30.0, 83.0)	62.0 (20.0, 84.0)	
Race, no. (%)				
American Indian	1 (0.9)	0 (0)	1 (0.4)	0.86
Asian	2 (1.7)	2 (1.7)	4 (1.7)	
Black	15 (12.8)	17 (14.3)	32 (13.6)	
Other	2 (1.7)	3 (2.5)	5 (2.1)	
White	97 (82.9)	97 (81.5)	194 (82.2)	
Ethnicity, no. (%)				
Hispanic	10 (8.5)	7 (5.9)	17 (7.2)	0.589
Not Hispanic	107 (91.5)	112 (94.1)	219 (92.8)	
Sex, no. (%)				
Female	32 (27.4)	26 (21.8)	58 (24.6)	0.406
Male	85 (72.6)	93 (78.2)	178 (75.4)	
Tobacco use, no. (%)				
Current	6 (5.1)	9 (7.6)	15 (6.4)	0.506
Never	46 (39.3)	41 (34.5)	87 (36.9)	
Past, quit > 1 year ago	39 (33.3)	48 (40.3)	87 (36.9)	
Past, quit ≤ 1 year ago	26 (22.2)	21 (17.6)	47 (19.9)	
Alcohol use				
No	70 (59.8)	83 (69.7)	153 (64.8)	0.145
Yes	47 (40.2)	36 (30.3)	83 (35.2)	
BMI				
Mean (SD)	26.5 (5.89)	26.4 (6.49)	26.4 (6.19)	0.903
Median (min, max)	25.2 (16.7, 56.4)	25.5 (2.92, 47.9)	25.3 (2.92, 56.4)	
Residence, no. (%)				
	12 (10.3)	8 (6.7)	20 (8.5)	0.623
City/urban	35 (29.9)	41 (34.5)	76 (32.2)	
Country/rural/small town	22 (18.8)	26 (21.8)	48 (20.3)	
Suburban	48 (41.0)	44 (37.0)	92 (39.0)	
Cancer site, no. (%)				
HNC of unknown primary	8 (6.8)	5 (4.2)	13 (5.5)	0.098
Larynx	19 (16.2)	35 (29.4%)	54 (22.9)	
Major salivary glands	6 (5.1)	2 (1.7%)	8 (3.4)	
Oral cavity	57 (48.7)	45 (37.8%)	102 (43.2)	
Paranasal sinuses	2 (1.7)	2 (1.7%)	4 (1.7)	
Pharynx	25 (21.4)	30 (25.2%)	55 (23.3)	
Cancer surgery, no. (%)				
No	8 (6.8)	14 (11.8)	22 (9.3)	0.281
Yes	109 (93.2)	105 (88.2)	214 (90.7)	
Type of head neck cancer surgery				
Biopsy only, no. (%)				
No	64 (54.7)	61 (51.3)	125 (53.0)	0.69
Yes	53 (45.3)	58 (48.7)	111 (47.0)	
Laryngectomy, no. (%)				
No	107 (91.5)	111 (93.3)	218 (92.4)	0.777
Yes	10 (8.5)	8 (6.7)	18 (7.6)	
Glossectomy, no. (%)				
No	101 (86.3)	104 (87.4)	205 (86.9)	0.96
Yes	16 (13.7)	15 (12.6)	31 (13.1)	
Tonsillectomy, no. (%)				
No	108 (92.3)	113 (95.0)	221 (93.6)	0.57
Yes	9 (7.7)	6 (5.0)	15 (6.4)	
Neck dissection, no. (%)				
No	62 (53.0)	70 (58.8)	132 (55.9)	0.441
Yes	55 (47.0)	49 (41.2)	104 (44.1)	
Current trach, no. (%)				
No	100 (85.5)	110 (92.4)	210 (89.0)	0.133
Yes	17 (14.5)	9 (7.6)	26 (11.0)	
Current PEG, no. (%)				
No	84 (71.8)	89 (74.8)	173 (73.3)	0.709
Yes	33 (28.2)	30 (25.2)	63 (26.7)	
Chemotherapy, no. (%)				
No	38 (32.5)	31 (26.1)	69 (29.2)	0.346
Yes	79 (67.5)	88 (73.9)	167 (70.8)	
Radiation dose				
Mean (SD)	6250 (1560)	6570 (1050)	6410 (1340)	0.067
Median (min, max)	7000 (0, 7500)	7000 (0, 7350)	7000 (0, 7500)	
Unknown	2 (1.7)	3 (2.5)	5 (2.1)	
Time since radiation (days)				
Mean (SD)	283 (501)	254 (438)	268 (469)	0.653
Median (min, max)	135 (22.0, 3130)	111 (20.0, 2970)	124 (20.0, 3130)	
Unknown	8 (6.8)	2 (1.7)	10 (4.2)	

### 3.2 Time to Therapy Initiation

**FIGURE 2:**
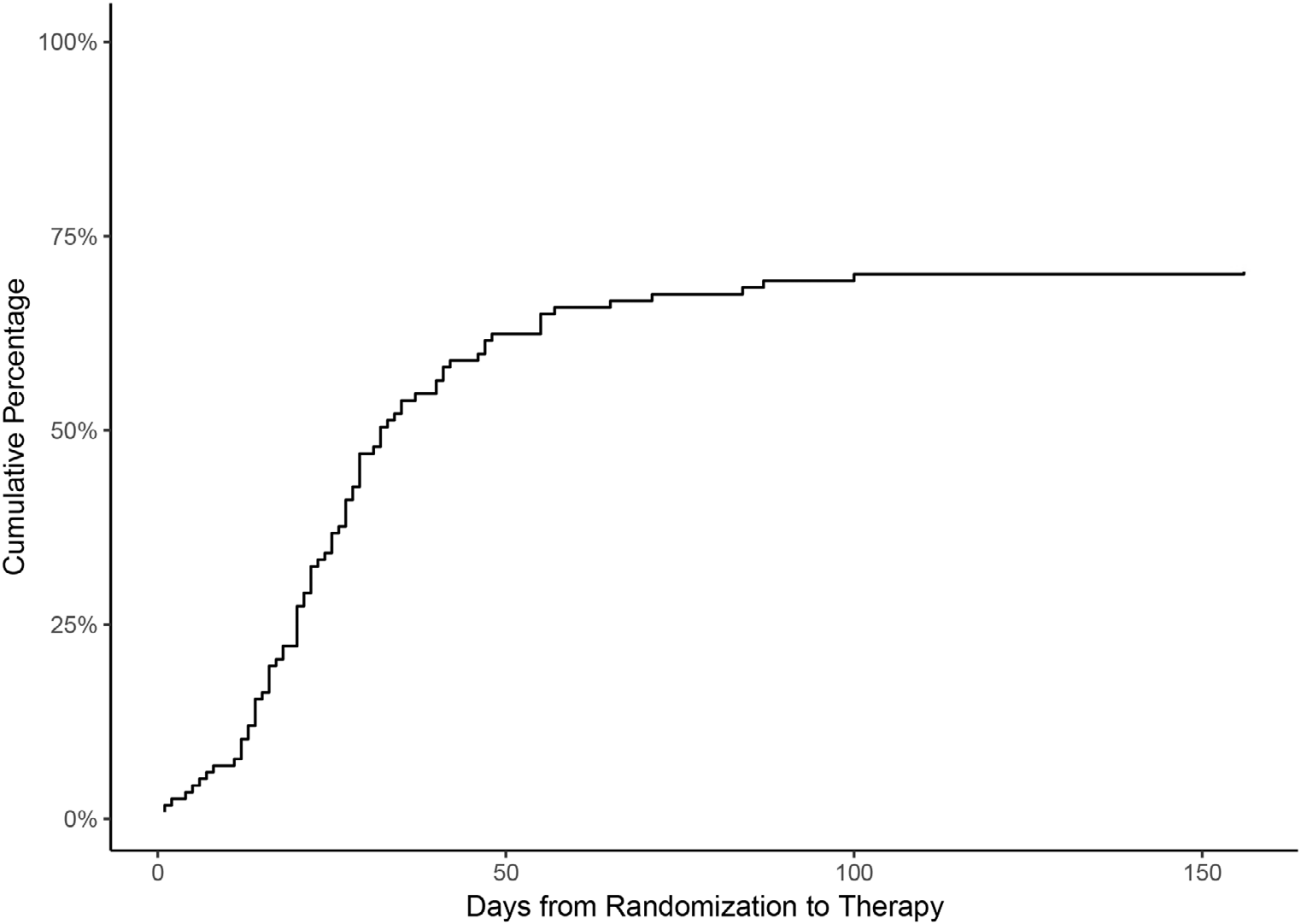
Time from randomization to initial therapy visit for participants who received usual care.

Of 117 participants in the Usual Care group, 83 (70.9%) initiated TGLT (Figure 2). Of those who initiated therapy, the average time from randomization to the first therapy visit was 29.8 days (SD 23.5 days). Of 119 APCD participants, 113 (94.9%) received the device. The average time from randomization to device acquisition was 17.86 days (SD 10.53 days).

### 3.3 Self‐Reported Adherence

Both groups self‐reported a high degree of adherence to their assigned treatment. The participants in the APCD group self‐reported an average of 6.0 (95% CI: 5.8–6.1) days of APCD use per week. Usual Care participants were advised to undertake a variety of self‐care activities potentially including self‐administered massage (average 5.7 days (95% CI: 5.4–5.9)), compression garment (average 4.7 days (95% CI: 4.3–5.2)), bandaging (4.6 days (95% CI: 3.9–5.2)), skin care regimens (6.2 days (95% CI: 5.9–6.5)), and exercises (5.4 days (95% CI: 5.2–5.7)).

### 3.4 Adverse Events

There were three adverse events involving two participants in the APCD group and both remained in the study. On participant experience cellulitis deemed possibly device related (Grade 3). A second reported claustrophobia and thyroiditis both deemed probably device related (Grade 3). There were no serious adverse events for either group.

### 3.5 Response to Therapy

#### 3.5.1 PRO Measures

**FIGURE 3:**
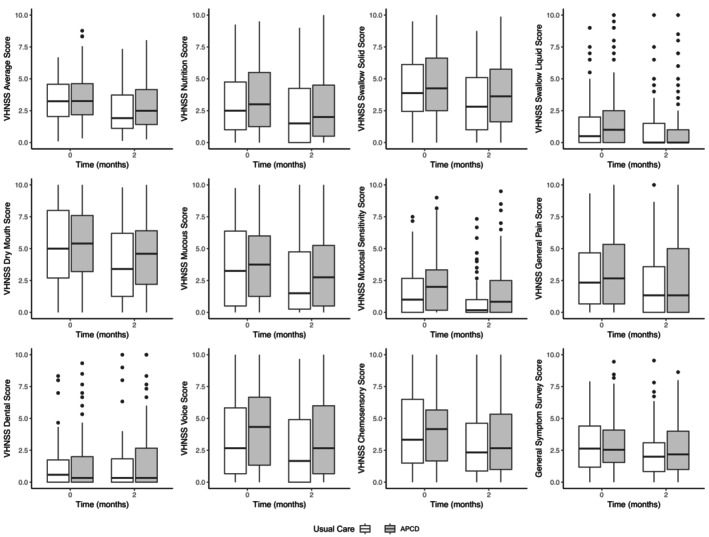
Patient reported outcomes at baseline and 2 months: Vanderbilt head and neck symptom survey version 2.0 plus general symptom survey.

**FIGURE 4:**
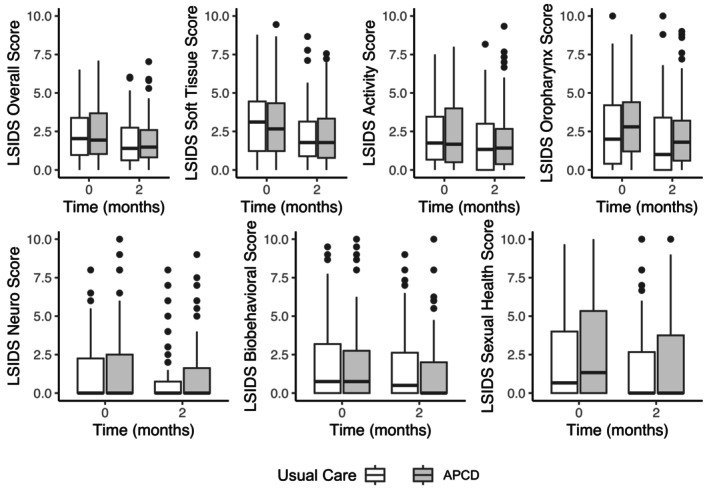
Patient reported outcomes at baseline and 2 months: Lymphedema symptom intensity and distress survey—head and neck version 2.0.

Symptom improvement was evident in both groups. No significant difference in the total score on the VHNSS or LSIDS was noted (Figures 3 and 4). Total VHNSS score was reduced by −0.37 ((95% CI: −0.61, −0.13), *p* = 0.003) in the Usual Care group and −0.27 ((95% CI: −0.52, −0.018), *p* = 0.036) in the APCD group. Total LSIDS score was reduced by −0.26 ((95% CI: −0.49, −0.04), *p* = 0.022) in the Usual Care group and −0.26 ((95% CI: −0.47, −0.04), *p* = 0.018) in the APCD group. Statistically significant improvements were noted on six of the subscales: three subscales in the APCD group and three in the Usual Care group. The average decrease in the scores for each subscale for Usual Care and the APCD are as follows: VHNSS pain subscale (Usual Care: −0.27 ((95% CI: −0.64, 0.09), *p* = 0.136); APCD: −0.38 ((95% CI: −0.76, 0.00), *p* = 0.050)), VHNSS solid swallowing subscale (Usual Care: −0.45 ((95% CI: −0.79, −0.11), *p* = 0.011); APCD: −0.27 ((95% CI: −0.63, 0.08), *p* = 0.136)), VHNSS liquid swallowing subscale (Usual Care: −0.18 ((95% CI: −0.49, 0.12), *p* = 0.244); APCD: −0.20 ((95% CI: −0.53, 0.12), *p* = 0.233)), VHNSS mucosal sensitivity subscale (Usual Care: −0.37 ((95% CI: −0.63, −0.11), *p* = 0.005); APCD: −0.33 ((95% CI: −0.63, −0.3), *p* = 0.032)), LSIDS soft tissue subscale (Usual Care: −0.38 ((95% CI: −0.67, −0.11), *p* = 0.007); APCD: −0.36 ((95% CI: −0.63, −0.10), *p* = 0.008)), and LSIDS neurologic subscale (Usual Care: −0.16 ((95% CI: −0.44, 0.12), *p* = 0.250); APCD: −0.17 ((95% CI: −0.46, 0.13), *p* = 0.275)).

#### 3.5.2 CRO Measures

**FIGURE 5:**
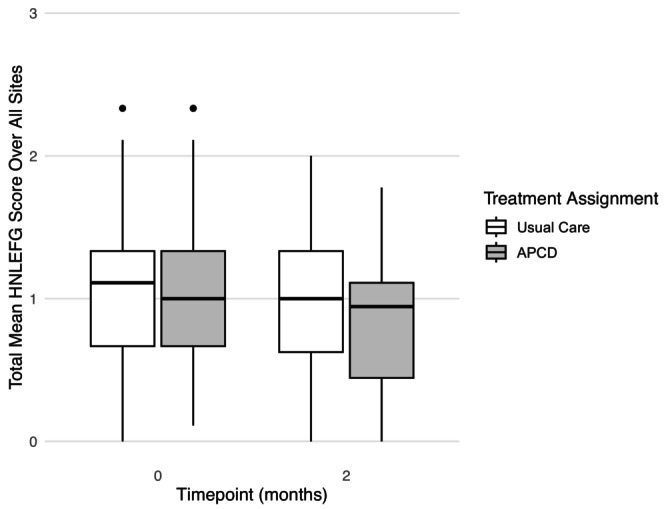
External lymphedema as measured by the Head and Neck Cancer Lymphedema and Fibrosis Grading (HN‐LEFG) criteria.

Digital photography: Participants in the APCD group experienced statistically significant improvement in swelling as evidenced by a reduction in the proportion of sites manifesting swelling (Figure 5). In the Usual Care group, the proportion of sites manifesting swelling decreased, but this did not reach statistical significance (APCD: 0.033 (95% CI: 0.017, 0.049), *p* < 0.001; UC: 0.017 (95% CI: −0.003, 0.036), *p* = 0.14). A comparison between the two groups at 2 months did not reach statistical significance (0.016 (95% CI: −0.006, 0.037), *p* = 0.159).

HN‐LEFG: Participants in the APCD group experienced a statistically significant improvement in swelling over the 2‐month period as evidenced by a reduction in the total HN‐LEFG score −0.081 (95% CI: −0.122, −0.041), *p* < 0.001. In the Usual Care group, the observed reduction in HN‐LEFG score over the 2‐month period was marginal −0.044 (95% CI: −0.088, 0.001), *p* = 0.057. The mixed‐effects model adjusting for baseline score and study site demonstrated a statistically significant difference in favor of the APCD group −0.085 (−0.152, −0.016), *p* = 0.016. (Figure 5).

Modified Patterson Scale: The Total Score at baseline was not significantly different. Site specific analyses of baseline values did not suggest any significant differences between groups. At 2 months, both groups showed improvement; however, the distribution was not significantly different between the two treatment groups (Total Score *p* = 0.903). There were, however, statistically significant differences observed at the floor of mouth and soft palate sites (*p* = 0.040, 0.042, respectively) favoring the APCD.

#### 3.5.3 Imaging

CT Imaging: Epiglottic thickness (UC: *p* = 0.91; APCD: *p* = 0.49) and prevertebral soft tissue thickness as measured on CT images was not reduced in either group (UC: *p* = 0.99; APCD: *p* = 0.41).

## 4 Discussion

This is the largest prospective, randomized trial conducted in HNCS associated lymphedema treatment reported to date. The study provides rich short‐term data on two interventional approaches: an APCD and Usual Care. Participants had previously untreated lymphedema, at least one lymphedema associated symptom of ≥ 4/10 severity and objective evidence of lymphedema on exam or imaging. Thus, this represents a population that would routinely be referred for lymphedema therapy. As there is no “gold standard” measurement tool to assess the extent of lymphedema or response to therapy in HNCS, we utilized a range of measurement tools including PROs, CROs, and imaging to capture salient outcomes. CROs demonstrated a statistically significant but modest benefit for the APCD as measured by external and internal soft tissue swelling. There was a similar decrease in lymphedema associated symptom burden and internal soft tissue swelling in both study groups. Therefore, our results provide confirmation of the short‐term (2‐month) effectiveness of the APCD for HNCS with treatment naïve lymphedema similar in magnitude to that seen in with TGLT.

Retrospective studies conducted in the general rehabilitation population demonstrate high rates of failure to initiate or complete ordered therapy and even lower rates of adherence to recommended home exercise programs [26, 27, 28, 29]. Similar findings have been noted in HNCS referred for lymphedema therapy [7, 10, 11, 12, 13]. Our study, which provides prospective data on TGLT in HNCS in both academic and community settings, confirms the retrospective data. Of 117 participants randomized to Usual Care, only 71% received treatment compared to 94.9% of those in the APCD group. Critically, of those who received Usual Care, a significant percentage experienced a delay in therapy initiation. The average time to the first therapy visit was 29.8 days (23.5 days SD). Our data underscores the challenges of initiating TGLT in a timely and efficient manner in the current barrier laden environment. Frequently cited systems barriers to initiating and completing lymphedema care include a fragmented referral system, lack of insurance coverage, and lack of availability of trained therapists [7, 10, 11, 12, 13]. Access to therapy is particularly problematic in rural areas resulting in increased travel time and expense for patients [30]. In addition, patient‐related barriers to completing the prescribed course of lymphedema therapy include high co‐pays, travel restrictions, time limitations, scheduling challenges, lack of perceived benefit, and poor performance status secondary to persistent symptoms such as fatigue and weakness [7, 10, 11, 12, 13].

The APCD may address some of these barriers. The device was developed to mimic the compression and massage techniques of therapist administered MLD thus providing convenient and consistent compression in the home setting [31]. Convenience is vital for patients or caregivers with time limitations or patients with symptoms such as fatigue and generalized weakness. Consistent and effectual self‐MLD correlates with response to therapy [32, 33]. Unfortunately, self‐MLD techniques are a challenge for many patients, particularly those with physical or cognitive impairment. In addition, fidelity may decline over time resulting in ineffectual self‐care [34]. Conversely, the donning process for the APCD is straightforward and lends itself to procedural uniformity.

In the breast cancer population, early detection and treatment of lymphedema results in improved clinical outcomes, decreased hospitalizations, and decreased health care costs [35]. A prospective surveillance and prevention model is emerging as standard of care in this population [36]. The NCCN Guidelines recommend baseline arm measurement followed by routine surveillance in patients at risk for arm lymphedema, and a prospective model with screening every 3 months noted decreased direct treatment costs [37, 38]. This allows early identification and intervention for subclinical lymphedema, thus potentially preventing the progression to chronic lymphedema with its associated symptoms and functional impairment. Historically, early identification of lymphedema in the HNCS population has been challenging due in part to measurement issues and lack of awareness of the need for early referral for lymphedema therapy. Baseline data demonstrated mild to moderate lymphedema in most participants, indicating that available measurement tools can identify early‐stage lymphedema in the HNCS population. Furthermore, most participants were responsive to both therapeutic approaches, supporting the concept that early‐stage lymphedema is responsive to therapy. Unfortunately, late‐stage lymphedema, with its associated fibrosis, is notoriously treatment refractory and remains therapeutically challenging. Despite the early‐stage lymphedema of our study population, the significant decrease in the HN‐LEFG score of the APCD group speaks to the potential value of early APCD therapy.

While both the APCD and TGLT are effective for management of lymphedema, a hybrid approach may optimize therapeutic outcomes by mitigating barriers or limitations encountered by each technique individually. The APCD may enable patients to initiate lymphedema treatment promptly while waiting for access to a lymphedema therapist. It may also help maintain gains between therapy sessions and enhance fidelity to a home self‐care routine. TGLT provides the setting for a therapeutic alliance and individualized care for the patient and their unique presentation. Therapists provide education, adjunctive therapeutic measures (such as bandaging), and instructions for home exercise and self‐care programs.

### 4.1 Strengths and Limitations

While the APCD is effective, it has limitations. Insurance coverage and time to device delivery are variable outside of a clinical trial. The device provides overall good coverage of involved tissues. However, some participants had lymphedema in areas that are not covered by the garment: the device could not effectively treat these sites. A small number of patients also report discomfort while wearing the device or poor garment fit.

Determination of lymphedema treatment response is a challenge in the HNCS population. To address known measurement issues, we looked for convergent results using a variety of tools including PRO's, CRO's, and radiographic imaging.

Adherence with TGLT and lymphedema self‐care is a major determinant of outcome. We collected self‐reported APCD and TGLT adherence. Unfortunately, self‐reported adherence data may be unreliable.

Given the mild to moderate severity of lymphedema in the study population, we are unable to speak to the generalizability to severe lymphedema. However, our prior study in participants with therapy refractory lymphedema found the APCD to be effective as compared to control despite the high level of lymphedema symptom burden and severity.

## 5 Conclusions

The APCD is an effective treatment modality for lymphedema in HNCS that addresses known barriers to TGLT. Both the APCD and TGLT can improve lymphedema in HNCS; a hybrid approach may be complimentary and optimize patient outcomes.

## Author Contributions

Data access: Dr. Derek K. Smith had full access to all of the data in the study and takes responsibility for the integrity of the data and the accuracy of the data analysis. Concept and design: Barbara A. Murphy, Sheila H. Ridner, and Derek K. Smith. Acquisition, analysis, or interpretation of data: Joseph M. Aulino, Barbara A. Murphy, Sheila H. Ridner, and Derek K. Smith. Critical revision of the manuscript for important intellectual content: Barbara A. Murphy, Sheila H. Ridner, Derek K. Smith, Cristina M. Kline‐Quiroz, Katrina M. Jensen, Ammar W. Sukari, Mihir K. Bhayani, Vikas Mehta, Harry Quon, Jennifer L. Shah, Christopher D. Willey, Neal E. Dunlap, Hoon K. Lee, and Joseph M. Aulino. Statistical analysis: Derek K. Smith. Obtained funding: Barbara A. Murphy and Sheila H. Ridner. Administrative, technical, or material support: Barbara A. Murphy and Sheila H. Ridner. Imaging Review: Joseph M. Aulino. Supervision: Barbara A. Murphy and Sheila H. Ridner.

## Funding

Barbara A. Murphy, MD—Research funding Tactile Medical. Derek Smith, DDS, PhD—Research funding Tactile Medical. Cristina Kline‐Quiroz, DO—Research funding Tactile Medical. Katrina Jensen M.A., CCC‐SLP—Research funding Tactile Medical. Ammar Sukari, MD—Research funding Tactile Medical. Mihir_Bhayani, MD—Research funding Tactile Medical. Research funding Tactile Medical. Vikas Mehta, MD—Research funding Tactile Medical. Harry Quon, MD—Research funding Tactile Medical. Jennifer Shah, MD—Research funding Tactile Medical. Christopher Willey, MD, PhD—Received funding from AACR‐Novocure, Varian Medical Systems, OMS Foundation, Mureva, Tactile Medical and has been a consultant and/or received honorarium from EMD Serono, LifeNet Health, and Guidepoint Global. Neal Dunlap, MD—Research funding Tactile Medical. Hoon Lee, MD—Research funding Tactile Medical. Joseph Aulino, MD—Research funding Tactile Medical. Sheila H. Ridner, PhD—Research funding Tactile Medical. The funder consulted with Drs. Murphy and Ridner on the protocol and design. The funder also provided advanced compression devices, web‐based support to complete data collection, collection management, on‐site fidelity monitoring, and review of the completed manuscript for factual content. The funder had no role in participant recruitment or consent, analysis, interpretation of the data, and the decision to submit the manuscript for publication.

## Ethics Statement

This study was completed in accordance with the World Medical Association Declaration of Helsinki (version 2002). The study was independently reviewed and approved by Institutional Review Boards at each site and by Scientific Review Committees at the designated Comprehensive Cancer Centers. Study activities were undertaken after confirming participant understanding and obtaining written informed consent.

## Conflicts of Interest

The authors declare no conflicts of interest.

## Data Availability

Researchers wishing to request study data can contact clinicalresearch@tactilemedical.com.
